# Linking complex disease and exposure data—insights from an environmental and occupational health study

**DOI:** 10.1038/s41370-022-00428-7

**Published:** 2022-03-28

**Authors:** Cataia Ives, Huaqin Pan, Stephen W. Edwards, Mark Nelms, Hannah Covert, Maureen Y. Lichtveld, Emily W. Harville, Jeffrey K. Wickliffe, Wilco Zijlmans, Carol M. Hamilton

**Affiliations:** 1 RTI International, Research Triangle Park, NC USA; 2 School of Public Health, University of Pittsburgh, Pittsburgh, PA USA; 3 Tulane University School of Public Health and Tropical Medicine, New Orleans, LA USA; 4 School of Public Health, University of Alabama at Birmingham, Birmingham, AL USA; 5 Faculty of Medical Sciences, Anton de Kom University of Suriname, Paramaribo, Suriname

**Keywords:** Epidemiology, Health studies, Child exposure/health, Children’s health, Vulnerable populations

## Abstract

The disparate measurement protocols used to collect study data are an intrinsic barrier to combining information from environmental health studies. Using standardized measurement protocols and data standards for environmental exposures addresses this gap by improving data collection quality and consistency. To assess the prevalence of environmental exposures in National Institutes of Health (NIH) public data repositories and resources and to assess the commonality of the data elements, we analyzed clinical measures and exposure assays by comparing the Caribbean Consortium for Research in Environmental and Occupational Health study with selected NIH environmental health resources and studies. Our assessment revealed that (1) environmental assessments are widely collected in these resources, (2) biological assessments are less prevalent, and (3) NIH resources can help identify common data for meta-analysis. We highlight resources to help link environmental exposure data across studies to support data sharing. Including NIH data standards in environmental health research facilitates comparing and combining study data, and the use of NIH resources and adoption of standard measures will allow integration of multiple studies and increase the scientific impact of individual studies.

## Introduction

Environmental epidemiological studies collect a variety of data, including both survey and exposure data, on environmental exposures and associated health outcomes. A systematic review of studies on barriers to public health data sharing identified a lack of standard protocols for data collection across studies as an important technical obstacle to translating research findings into public health interventions [1]. This analysis examines the use of common exposure measures across selected National Institutes of Health (NIH)-funded environmental epidemiologic studies and provides recommendations to promote the utility of environmental epidemiologic studies by advancing the use of standard protocols, particularly those focused on examining exposures to chemical and non-chemical stressors.

The rationale for our analysis is multi-pronged. First, statistical power and scientific efficiency would be maximized by combining data from multiple studies and thus increasing their effect size, but standardizing the various environmental exposure data collection measurement protocols and linking similar variables is a fundamental challenge [2].

Second, environmental health research relies on data-driven semantic standards for exposure science, centered on characterizing the interactions of a receptor (i.e., an individual or human population) with one or more environmental chemical and non-chemical stressors [3].

Third, exposure data may be expensive to collect, and studies may evaluate exposures in ways that can be difficult to combine across studies. The field of exposure science has identified the need to link information on toxicity to real-world outcomes and to use exposure data for chemical prioritization.

Environmental exposures are complex, encompassing a variety of domains and study types. The “exposome” concept, defined as the totality of exposures experienced by an individual during their life and the health impacts of those exposures [4], strives to capture the diversity and range of ecosystem, social, physical, chemical, and lifestyle exposures [5]. The complexity of exposome data and the need to increase the scale of exposome studies require data homogeneity to allow data merging and integration. The public health exposome represents a further refinement of the original exposome concept, integrating exogenous and endogenous exposures across the lifespan [6]. For environmental epidemiological studies, which typically include exposure and survey data, standards for annotating and identifying common data elements promote data reuse [7]. Therefore, establishing and adopting common data elements makes more data sources available for data integration using analysis and fusion models [8].

### NIH environmental health resources

To assess the use of environmental exposures, we compiled several NIH resources relevant to environmental exposures to assess the potential impact of using standard measures. We chose the following resources because they contain substantial environmental health study data or measures, providing clear opportunities to link study variables and support collaborative analyses.

**Children’s/Human Health Exposure Analysis Resources (CHEAR/HHEAR)** are centralized networks of exposure analysis tools, services, and expertise to support NIH-funded researchers studying human health [9]. The HHEAR Data Center (https://hheardatacenter.mssm.edu/) provides a repository of data and laboratory analysis results for CHEAR and HHEAR studies and provides statistical and data analysis services to external researchers.

NIH’s  **Environmental Influences on Child Health Outcomes (ECHO)** is one of the largest environmental health programs in terms of outcome and exposure measures and number of participants [10]. ECHO supports multiple longitudinal studies using existing study populations to investigate environmental exposures—including physical, chemical, biological, social, behavioral, and natural exposures and the effects of built environments—on child health and development [11]. The ECHO-wide Cohort Data Collection Protocol includes assessments of a rich set of environmental exposures, which can serve as a resource for environmental health research [12].

 **PhenX (consensus measures for Phenotypes and eXposures)** is an NIH common data elements project driven by the research community. It provides tools to help investigators incorporate recommended measurement protocols into their studies (https://www.phenxtoolkit.org/). PhenX contains standard measures across 29 research domains to date, including survey questionnaires, clinical examination, medical records abstraction, and bioassay protocols. The PhenX Environmental Exposures domain covers such topics as residential and occupational history, early-life exposures, environmental contaminants, and specific sample collections, providing a useful starting framework of standard measures to investigate environmental contributors to complex diseases [13].

The  **Database of Genotypes and Phenotypes (dbGaP)** is a public data repository for a variety of NIH-funded studies, including genotype and phenotype data (https://www.ncbi.nlm.nih.gov/gap/) [14]. Currently, it hosts more than 1800 studies from 22 NIH institutes and centers, including 15 studies from National Institute of Environmental Health Sciences (NIEHS). As one of the largest public data repositories, studies in dbGaP have traditionally focused on genetic associations with human diseases and conditions but have recently included exposure data.

## Methods

### Assess environmental exposure measures among NIH resources

We compiled environmental exposure measures from four NIH resources (ECHO, CHEAR/HHEAR, PhenX, and dbGaP) to identify overlap and gaps. Exposure measures were differentiated by mode of collection into “Environmental Assessment” (e.g., “Pets in Household”) and “Biological Assessment” (e.g., “PAHs”) groups. Here, *environmental assessments*, such as “Characteristics of Current Residence,” refer to direct exposures as measured by interviewer-administered, self-report, or laboratory analysis protocols. *Biological assessments*, such as “Biomarker of exposure to nicotine-containing products—Urine,” refer to chemical assays correlated with specific environmental exposures.

The four resources used different environmental assessment categories, which were harmonized for this analysis. We used CHEAR/HHEAR study descriptions from the data center [9] and classes in the HHEAR Ontology (http://purl.bioontology.org/ontology/HHEAR) to annotate the HHEAR studies to exposure categories (see Supplementary Table S1 for detail). We then used keywords extracted from PhenX environmental exposure protocols to map ECHO data elements. We used the dbGaP Advanced Search feature to identify ECHO environmental assessments present in dbGaP studies. Buckley et al. described existing or planned chemical biomonitoring in either ECHO mother or child cohorts for 15 broad chemical classes, including both well-characterized and emerging chemicals [10]. Using these biological assessments from ECHO and keywords extracted from PhenX environmental exposure protocols, we searched the dbGaP [14] data repository. Biological assessments from ECHO were also mapped to HHEAR analytes, which are targeted analyses assessed by HHEAR Lab Hubs [9].

### Comparison between CCREOH and ECHO studies’ exposure measures

We compiled the clinical measures and exposure assays from the Caribbean Consortium for Research in Environmental and Occupational Health (CCREOH) study to compare them with those in the ECHO-wide Data Collection Protocol [15]. We conducted mapping between CCREOH health assessments for topics such as health history, depression, perceived stress, and exposure history [15] and ECHO-wide Cohort Data Collection Protocols from the ECHO project site [12]. To identify exposures measured, we conducted mapping between ECHO chemical assays and CCREOH biomarkers.

## Discussion

### Assessment of environmental exposure measures in NIH resources

**Fig. 1:**
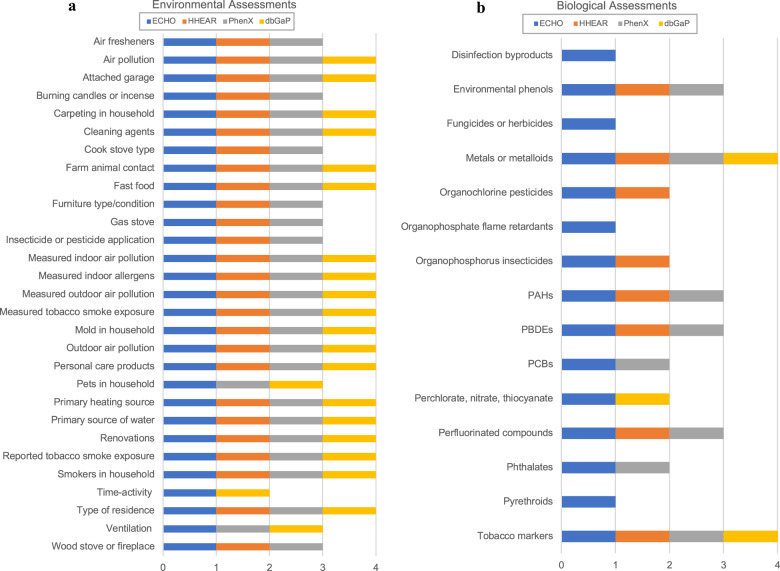
Presence of environmental exposures in four community resources. **a** Environmental assessments and **b** biological assessments.

In this study, we compiled environmental exposure measures and compared their presence in four resources: ECHO, CHEAR/HHEAR, PhenX, and dbGaP. Figure 1 shows environmental exposure measures from ECHO and compares their presence and absence in four NIH resources. There are a number of environmental assessments in dbGaP studies (Fig. 1a) and biological assessments in ECHO (Fig. 1b); however, biological assessment data are quite limited in other resources (see more details in the Supplementary Information).

Many environmental health studies include more survey data than environmental measures because environmental data are costly to collect, and statistical concerns regarding small effect sizes further hamper comprehensive environmental epidemiologic study designs. Given these key challenges, using resources to link with other data sets becomes invaluable because it helps individual studies find additional environmental data to enhance their statistical power for hypothesis testing. Creating linkages among measures from PhenX, ECHO, HHEAR, and dbGaP makes it easier to identify similarities and differences among exposure categories in the four resources. Individual environmental health studies such as CCREOH could leverage these NIH resources to identify study data sets for comparative analysis. For example, seven ECHO cohorts collected metals or metalloids assay data [16], corresponding with eight HHEAR analytes, 5 PhenX protocols, and 33 dbGaP studies that collected metals or metalloids (Table S2). This approach provides a benchmark for use of environmental exposure measures in NIH-funded research and potentially serves as a framework for standard environmental exposure measures for prospective studies and data harmonization with retrospective studies, both types of which are present in ECHO. This analysis demonstrates a possible approach to combining data from different but conceptually related studies.

### Opportunities for increasing CCREOH study impact

We chose existing studies that provided clear opportunities for linking study variables and that would support collaborative analysis of cross-cutting research questions related to environmental exposures, human health, and disease. We first examined the studies published by the NIEHS Deepwater Horizon Research Consortia [17]. Cross-consortia investigator groups focused on commonalities across environmental exposures (fish), epidemiologic data, and resilience measures, the latter resulting in a resilience framework [18]. Overall, a dominance of study-specific measures limited the use of common data elements across the consortia studies [2]. Although study-specific measures are necessary and valuable to address local community concerns and specific research questions, standard measures facilitate the ability to combine data from conceptually related studies. We then continued the assessment with another study, the CCREOH Cohort Study, funded by the NIH Fogarty International Center [15]. CCREOH was selected as a good use case as a cohort that could benefit from collaboration with investigators who used similar data collection protocols. The study is a longitudinal follow-up of pregnant women (*N* = 1143) and their children from birth to 48 months (*N* = 992), providing several timepoints to assess birth and neurodevelopmental outcomes linked to environmental health exposures [15], including biospecimen samples for chemical exposure analysis and non-chemical stressors assessment.

#### CCREOH and ECHO share health measures

Mapping between CCREOH health assessments and ECHO-wide data collection protocols resulted in five common instruments and ten different instruments between the two studies, with some overlapping variables (Table 1). CCREOH assessments included assessments of depression and perceived stress administered prenatally; neurodevelopmental assessments, including the Bayley Scales of Infant Development for assessment of infant cognitive and motor development at 12–27 months; assessments of cognitive and social-emotional development at 36 months; and planned assessments of executive function at 48 months [15]. This partial overlap of identical and alternative instruments used for the same data element concept presents opportunities for meta-analysis for some data elements, and challenges for harmonization for other data elements, with the ECHO study data across child life stages [11].

**Table 1:** CCREOH and ECHO share standard questionnaire measures with some collected using the same instruments and others with different instruments.

CCREOH instruments	ECHO instruments
Modified Checklist for Autism in Toddlers	Same
Child Behavior Checklist	Same
Bayley Scales of Infant and Toddler Development	Same
Edinburgh Depression Scale	Same
Cohen Perceived Stress Scale	Same
Alcohol, Smoking and Substance Involvement Screening Test V3.0	Maternal Medical Record Abstraction, Youth Risk Behavior—Substance Use
Brief Trauma Interview	Childhood Trauma Questionnaire
Child Development Review	Ages and Stages Questionnaire
Exposure History Form	Household Chemical Exposure; Household Exposure to Secondhand Smoke
Dietary Questionnaire	NCI Diet History Questionnaire Third Edition (DHQ III); Dietary Screener Questionnaire—Self Report; Block Questionnaire; Dietary Screener Questionnaire
General Health and Demographic Questionnaire	Various Health and Demographic Questionnaires
Generation R Questionnaire (for 36 months)	Various Health Questionnaires
Prenatal Life Events Scale	Crisis in the Family Systems—Revised (CRISYS-R); Stressor Checklist
SF 36 Health Survey	Perceived Stress Scale
Social Support List	PROMIS v2.0 - Emotional Support; Informational Support; Instrumental Support

#### CCREOH and ECHO share biological assessments

ECHO collected essential biospecimens in mothers and children across a broad range of life stages, from prenatal to adolescence [12], whereas biological samples in CCREOH were collected in mothers during the first/second and third trimester of pregnancy; at birth; and in children at 12 and 36 months of age, with planned collection at 48 months [15]. ECHO survey modules measured biomarkers of 15 broad chemical classes of interest [10]. CCREOH and ECHO shared biomarkers indictive of specific biological assessments, as shown in Table 2. This data set, with additional planned chemical assays, aligns well with the 4 to 34 ECHO cohorts with neurodevelopmental assessments and assays for the four chemical groups [16]. Telomere length was also an assessment conducted by both ECHO and by CCREOH that was collected via maternal and child buccal swab [15].

**Table 2:** CCREOH and ECHO share biomarkers indicative of specific biological assessments.

ECHO chemical assays	CCREOH analyzed (+in progress)
Metals/metalloids (Pb, Hg, Cd, Mn)	400 (+600 women)
Herbicides	200 women
Organophosphorus insecticides	200 women
Pyrethroids	200 women
Telomere length	786 Children (+1033 women, 60 children)
Metabolomics	(336 women)

#### CCREOH study data could be enriched with ECHO cohort data

ECHO includes 69 cohorts consisting of existing data collected prior to the implementation of ECHO and new data to be collected using the ECHO-wide Cohort Data Collection protocol [19]. The existing data collection has a large sample size containing (1) a range of 4 to 34 cohorts that collected 21 neurodevelopmental assessments and assays for 12 chemical groups [16], (2) a range of 1 to 69 cohorts that collected the 47 measures of family environment data [11], and (3) 25,526 subjects with descriptive characteristics of mothers of singleton live births by gestational age at birth category [20]. The ECHO Program provides opportunities for potentially combining and comparing data analyses across up to 69 cohorts collecting data using one study protocol and published using the existing and new data [12, 19].

Furthermore, in its strategic plan for 2020–2024, ECHO aims to “make its data and biospecimens accessible to a wide scientific community, in forms that are (1) compatible with other data sets and (2) suitable for use in multiple analyses” [21]. ECHO data sets will become an even more valuable resource for the environmental health research community, and utilization of common data elements will enable further data integration, expanding the potential for meta-analysis and extending the impact from the original individual studies to elucidate subtle and complex interactions between environmental exposures and patient outcomes.

CCREOH provides a rich data set of outcome measures and biospecimens from mother/child dyads. CCREOH measures have a substantive overlap with the environmental exposure measurements and health outcomes in ECHO; therefore, we assessed the standard measures to identify opportunities for meta-analysis to increase statistical power or to validate findings at different geographic locations. Given the degree of overlap of chemical measures, CCREOH has the potential to serve as a highly feasible pilot for ECHO to develop with a process for integrating data from external studies as part of the ECHO collaboration process open to the environmental health research community.

## Conclusions

This assessment and analysis of the prevalence of and commonalities among environmental exposure measures demonstrates the need for standard measures for combining data from conceptually related resources. As indicated by the CCREOH study, the use of standard measures among studies increases opportunities for meta-analysis and may be used to validate findings across geographic locations. With standard measures, NIH resources may expand the potential of individual studies by enabling cross-study analysis, contributing to the body of knowledge on environmental health outcomes. Individual environmental health studies can use NIH resources to identify study data sets for opportunities for comparative analysis while preserving some flexibility for community- and culturally tailored inquiry and maintaining scientific rigor.

## Disclaimer

The views expressed are those of the authors and do not necessarily represent the official position of NIEHS.

## Supplementary informion

Supplemental information
