# Supplementary material for: Linking complex disease and exposure data—insights from an environmental and occupational health study
Source: J Expo Sci Environ Epidemiol. 2022 Mar 28;33(1):12–6. doi: 10.1038/s41370-022-00428-7 (PMC9515242; doi:10.1038/s41370-022-00428-7)
Supplement: Supplementary file 1 — Supplemental information [file 41370_2022_428_MOESM1_ESM.pdf]

LINKING COMPLEX DISEASE AND EXPOSURE DATA-INSIGHTS FROM AN ENVIRONMENTAL AND OCCUPATIONAL HEALTH STUDY

Cataia L. Ives, Huaqin Pan, Stephen W. Edwards, Mark Nelms, Hannah Covert, Emily W. Harville, Jeffrey K. Wickliffe, Wilco Zijlmans, Maureen Y. Lichtveld, and Carol M. Hamilton

Table S1. ECHO Environmental Assessment Data Elements in Major Resources. The number of HHEAR studies, PhenX protocols, and dbGaP studies corresponding to each ECHO environmental assessment data element is shown. HHEAR studies are studies available in the HHEAR Data Center. PhenX protocols are a standard data collection procedure, recommended by a PhenX Working Group. dbGaP studies are NIH studies hosted in the dbGaP data repository.

ECHO Environmental Assessment Data Element	HHEAR Studies	PhenX Protocols	dbGaP Studies
Air fresheners	2	4	0
Air pollution	9	2	2
Attached garage	11	4	1
Burning candles or incense	1	5	0
Carpeting in household	5	5	1
Cleaning agents	2	5	1
Cook stove type	11	1	0
Farm animal contact	2	3	2
Fast food	2	8	2
Furniture type/condition	7	4	0
Gas stove	4	1	0
Insecticide or pesticide application	7	5	0
Measured indoor air pollution	7	1	2
Measured indoor allergens	3	1	1
Measured outdoor air pollution	1	1	2
Measured tobacco smoke exposure	4	5	7
Mold in household	2	2	2
Outdoor air pollution	15	1	3
Personal care products	11	4	3
Pets in household	0	2	12
Primary heating source	4	3	4
Primary source of water	15	4	4
Renovations	18	3	4
Reported tobacco smoke exposure	7	14	160
Smokers in household	1	4	7
Time-activity	0	0	2
Type of residence	3	2	2
Ventilation	0	2	4
Wood stove or fireplace	5	4	0

Table S2. ECHO Biological Assessment Data Elements in Major Resources. The number of HHEAR analytes, PhenX protocols, and dbGaP studies corresponding to each ECHO assay group is shown. The ECHO Assay Group consists of a group of assays, or chemical class, collected in ECHO. HHEAR analytes are targeted analyses assessed by HHEAR Lab Hubs. PhenX protocols are a standard data collection procedure, recommended by a PhenX Working Group. dbGaP studies are NIH studies hosted in the dbGaP data repository.

ECHO Assay Group	HHEAR Analytes	PhenX Protocols	dbGaP Studies
Allergens	0	1	136
Disinfection byproducts	0	0	0
Environmental phenols	14	13	0
Fungicides or herbicides	0	0	0
Illicit drugs	3	5	95
Metals or metalloids	8	5	33
Organochlorine pesticides	5	0	0
Organophosphate/other flame retardants	0	0	0
Organophosphorus insecticides	1	0	0
Perchlorate, nitrate, thiocyanate	0	0	2
Perfluorinated compounds	8	1	0
Phthalates	0	5	0
Polybrominated diphenyl ethers (PBDEs)	2	1	0
Polychlorinated biphenyls (PCBs)	0	1	0
Polycyclic aromatic hydrocarbons (PAHs)	2	1	0
Pyrethroids	0	0	0
Tobacco markers	2	1	31
